# Investigation of Microstructure, Hardness, and Wear Behavior of Hardfacing Produced by Wire Laser Additive Manufacturing

**DOI:** 10.3390/ma19143003

**Published:** 2026-07-12

**Authors:** Natan Damian Crozetta, Andeson Daleffe, Pedro Henrique Menegaro Possamai, Henrique Cechinel Casagrande, Gilson de March, Paulo Eduardo Ceccacci de Lion

**Affiliations:** 1 Department of Additive Manufacturing, Centro Universitário SATC, Criciúma 88805-380, Brazil; natan.crozetta@satc.edu.br (N.D.C.); anderson.daleffe@satc.edu.br (A.D.); pedro.possamai@satc.edu.br (P.H.M.P.); paulo_eduardo_lion@hotmail.com (P.E.C.d.L.); 2 Department of Materials Engineering, Federal University of Santa Catarina (UFSC), Florianópolis 88040-900, Brazil; 3 Department of Mining, Metallurgical and Materials Engineering, Federal University of Rio Grande do Sul (UFRGS), Porto Alegre 91501-970, Brazil; gilson.march@satc.edu.br

**Keywords:** Wire Laser Additive Manufacturing (WLAM), DUR600, abrasive wear resistance

## Abstract

Wire Laser Additive Manufacturing (WLAM) has emerged as a promising alternative for the fabrication and repair of components subjected to severe wear conditions due to its high deposition rate, efficient material utilization, and localized thermal control. In this study, the WLAM process using DUR600 wire as the feedstock material for the deposition of abrasion-resistant coatings was investigated. The deposited specimens were characterized by optical emission spectroscopy (OES), X-ray diffraction (XRD), optical microscopy (OM), scanning electron microscopy coupled with energy-dispersive X-ray spectroscopy (SEM/EDS), Vickers microhardness testing, and dry sand/rubber wheel abrasion testing in accordance with ASTM G65. The deposits exhibited a predominantly martensitic microstructure with retained austenite, as confirmed by XRD. Hardness values ranged from 749 to 817 HV, with an average of 783 ± 18 HV, while the average volumetric loss in the abrasive wear test was 150.26 mm^3^. This behavior was attributed to the presence of the martensitic matrix and retained austenite, whose combined effect directly influences the tribological performance of WLAM coatings produced using DUR600 wire.

## 1. Introduction

Additive Manufacturing (AM) has become a strategic technology for producing complex metallic components through a layer-by-layer deposition process directly from digital models [1,2,3,4,5,6]. Compared with conventional subtractive manufacturing, AM offers reduced material waste, shorter production times, greater geometric flexibility, and the ability to tailor material properties for demanding service conditions [7,8,9,10]. These advantages provide greater design flexibility and improved manufacturing efficiency compared with conventional processes such as casting, forging, and machining [2].

Among the available AM technologies, Wire Arc Additive Manufacturing (WAAM) has attracted considerable attention owing to its high deposition rates and capability to fabricate large-scale components with complex geometries [11,12,13,14]. In addition, the process enables the use of a wide range of metallic materials, including carbon steels, low-alloy steels, stainless steels, aluminum alloys, nickel-based alloys, and titanium alloys. Multiple wire-feeding configurations, such as dual-wire and twin-wire systems, further enhance control over the chemical composition of the deposited material, enabling in situ alloy development and the fabrication of multi-material components with tailored mechanical, corrosion, and wear-resistant properties [15,16,17,18,19].

Laser-based additive manufacturing technologies have also gained increasing interest because of their high dimensional accuracy, precise thermal control, and excellent metallurgical quality [20,21]. Wire Laser Additive Manufacturing (WLAM) employs a focused laser beam to continuously melt a metallic wire feedstock delivered onto a substrate [3,22]. Compared with powder-fed systems, wire-fed processes offer higher deposition efficiency, near-complete material utilization, lower operational costs, reduced environmental contamination, and simplified feedstock handling [11,12,23,24,25].

WLAM has found growing applications in mining, steelmaking, oil and gas, aerospace, and tooling industries due to its high deposition rate and localized heat input, making it particularly suitable for manufacturing large components, repairing worn surfaces, and depositing wear-resistant coatings [2,7,26]. However, the rapid thermal cycles inherent to the process strongly influence solidification behavior and microstructural evolution, thereby affecting the mechanical and tribological performance of the deposited material [7,27,28].

Among hardfacing consumables, DUR600 has attracted attention because of its high hardness and the presence of carbide-forming alloying elements, particularly chromium and molybdenum [29,30,31,32,33]. These elements promote the formation of hard phases dispersed within the metallic matrix, enhancing abrasion and impact resistance while maintaining good weldability and metallurgical stability under severe service conditions [31,32,33,34,35].

The application of DUR600 wire in WLAM represents a promising approach for manufacturing and repairing wear-resistant components [36,37]. Nevertheless, the final performance of the deposited coating strongly depends on the interaction between processing parameters and microstructural evolution [7]. Variables such as laser power, travel speed, wire feed rate, and heat input directly affect bead geometry, substrate dilution, phase formation, carbide distribution, residual stresses, and hardness variations throughout the deposited layers [38,39,40,41,42,43,44,45]. Therefore, comprehensive microstructural and mechanical characterization is essential to understand the solidification mechanisms, phase transformations, and tribological behavior of WLAM deposits [46,47].

Understanding the relationship between WLAM processing parameters and microstructural evolution is essential because the transient thermal cycles imposed during layer-by-layer deposition govern solidification, grain growth, phase formation, and ultimately the mechanical performance of the manufactured components [48,49]. Previous studies have demonstrated that processing conditions significantly influence phase fractions, defect formation, microstructure refinement, and mechanical properties in WLAM-fabricated alloys [50,51,52]. Despite these advances, a scientific gap remains regarding the application of commercial wear-resistant consumables such as DUR600, particularly concerning the correlation between laser deposition parameters, microstructural evolution, hardening mechanisms, and tribological performance.

To characterize the coatings produced by WLAM using DUR600 wire, optical emission spectroscopy (OES), optical microscopy (OM), scanning electron microscopy coupled with energy-dispersive X-ray spectroscopy (SEM/EDS), X-ray diffraction (XRD), Vickers hardness testing, and dry sand/rubber wheel abrasion testing were employed to establish correlations among chemical composition, microstructure, and mechanical performance [33,34,35,53,54,55,56,57,58,59].

Therefore, this study investigates the application of WLAM using DUR600 wire for the deposition of hardfacing coatings intended to improve the wear resistance of components operating under severe abrasive conditions. Furthermore, it evaluates the influence of deposition parameters, substrate dilution, and microstructural evolution on the mechanical and tribological performance of the deposited material, providing technical guidance for the application of WLAM in surface engineering, industrial component repair, and the development of advanced wear-resistant coatings [7,34,35,37,59].

## 2. Materials and Methods

In this study, the Wire Laser Additive Manufacturing (WLAM) process was employed to fabricate specimens for abrasive wear testing using DUR600 welding wire with a diameter of 1.2 mm. The manufacturing system consisted of a three-axis Cartesian robotic platform, where the X, Y, and Z axes corresponded to the transverse, longitudinal, and vertical movements, respectively. The system was integrated with a high-power laser source coupled to the deposition unit, enabling precise control of the material deposition process.

### 2.1. DUR 600 Welding Wire According to DIN 8555 Standard

The deposition wire used in this study was DUR 600, classified according to the DIN 8555 standard [60], which establishes requirements for welding consumables applied to wear-resistant coatings. The consumable is supplied by the manufacturer UTP (Bad Krozingen, Germany) and has a nominal diameter of 1.2 mm, being widely used in deposition processes for applications subjected to severe abrasive wear.

According to the manufacturer’s specifications, the deposited material produced using DUR 600 wire exhibits a typical hardness in the range of 57 to 62 HRC, characterizing it as a high wear-resistant consumable for abrasive conditions. The figure also indicates the polarity condition adopted in the process, configured as direct current electrode positive (DC+), a setup commonly used to ensure greater arc stability and improved metal transfer efficiency during deposition.

The nominal chemical composition of the welding wire is not presented in this section and is organized separately in Table 1, in order to better systematize the material information used in this work.

### 2.2. Additive Manufacturing Equipment, Deposition Strategy, and Process Parameters

**Figure 1:**
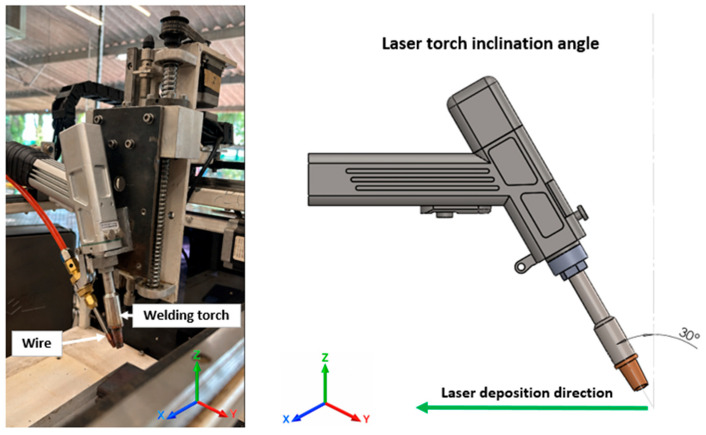
Wire Laser Additive Manufacturing (WLAM) system.

Computer Numerical Control (CNC) programming and process management were performed using Mach3 software (Version R3.042.029). The additive manufacturing equipment was manufactured in China and supplied in Brazil by Rohmes. The experimental setup adopted for the WLAM process is schematically illustrated in Figure 1.

A carbon steel plate measuring 12.7 × 25.4 × 76.2 mm was used as the substrate material for the deposition process. During additive manufacturing, the deposition head was positioned at an inclination angle of 30° relative to the deposition direction (Y-axis) to promote geometric stability and ensure uniform layer formation throughout the build.

Material deposition was achieved through the sequential overlapping of adjacent weld beads, producing continuous deposition tracks along the fabricated specimen and ensuring adequate interlayer fusion as well as dimensional uniformity. A parallel-track deposition strategy was adopted to improve heat distribution during the manufacturing process. A total of three successive layers were deposited, resulting in an overall build height of approximately 4 mm, with an average layer height of about 1.5 mm. This build height was intentionally selected considering the intended application of the deposited material as a wear-resistant coating for engineering components. An overall thickness of approximately 4 mm provides sufficient coating volume to maintain its protective performance under service conditions, ensuring adequate wear allowance and extending the operational lifetime of the component.

**Figure 2:**
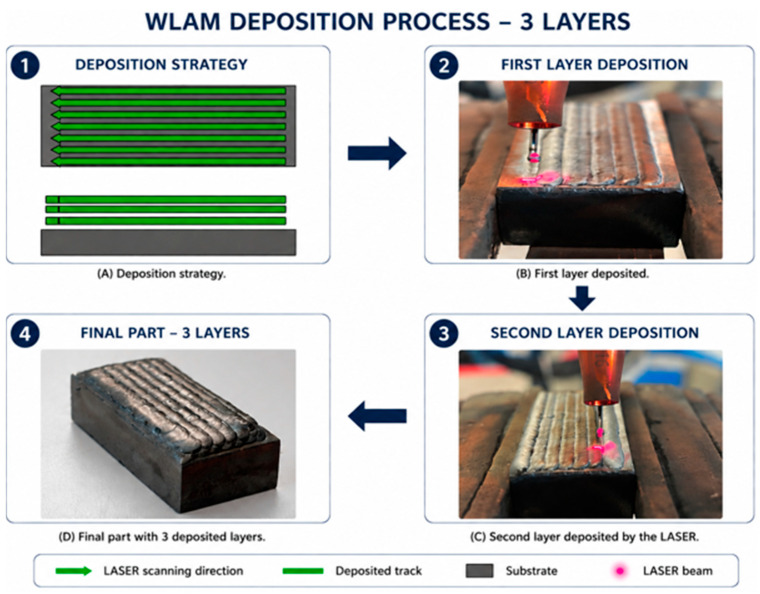
Deposition strategy (**1**), first deposited layer (**2**), second deposited layer (**3**), and final specimen with three deposited layers (**4**).

Furthermore, according to the literature [62], FeCrC hardfacing coatings produced by the Shielded Metal Arc Welding (SMAW) process are typically applied in a single layer, resulting in coating thicknesses comparable to those required for industrial wear-resistant applications. Since laser deposition generally produces thinner individual layers due to their lower dilution and higher dimensional control, three successive laser-deposited layers were employed in the present study to achieve a final coating thickness equivalent to that commonly obtained by conventional SMAW hardfacing. This strategy ensures representative coating geometry while enabling a more reliable comparison with conventional FeCrC hardfacing systems reported in the literature. Figure 2 details the deposition strategy employed in this study, as well as the fabrication of the deposited layers.

The WLAM processing parameters were optimized to achieve stable deposition conditions, adequate metallurgical bonding, and a high material deposition rate. As summarized in Table 2, the additive manufacturing process was carried out using a laser power of approximately 1500 W, a wire feed rate of 103 cm/min, a travel speed of 200 mm/min, and a shielding gas flow rate of 8 L/min with high-purity argon. Under these conditions, the estimated heat input was approximately 450 J/mm (Equation (1)), providing sufficient energy to ensure proper fusion and interlayer adhesion while minimizing geometric distortion and preserving the dimensional stability of the deposited structure. The heat input was calculated according to Equation (1).

(1) $$HI=\eta \frac{P}{v}$$

HI = (Heat Input): expressed in Joules per millimeter (J/mm).

η = (Thermal Efficiency/Process Efficiency)

*P* (Power): expressed in Watts (W)

*v*: (Travel Speed/Welding Speed): expressed in millimeters per second (mm/s)

$$HI=1\;\frac{1500\;(W)}{3.33333(\frac{mm}{s})}=450\;J/mm$$

The selection of these parameters was based on preliminary process stability studies and on achieving continuous beads with adequate filler material fusion and good adhesion to the substrate. As reported in the literature, laser power, travel speed, and wire feed rate are the primary parameters responsible for controlling the heat input in laser deposition processes, directly influencing the melt pool geometry, deposition rate, and the quality of the deposited material [63,64]. The parameters adopted in this study provided suitable conditions for the fabrication of the coatings, minimizing macroscopic defects and ensuring the dimensional stability of the produced samples.

The average deposition time per bead was approximately 20 s, while the total effective manufacturing time was about 7 min, highlighting the potential of the WLAM process for the rapid fabrication of medium-sized metallic components. A relatively short interpass time was also observed between successive layers, a condition that may directly influence the cumulative thermal cycles experienced during manufacturing and, consequently, the microstructural evolution of the deposited material.

Following fabrication, no heat treatment or additional post-processing operations were applied to the manufactured specimens. In order to evaluate the chemical composition, microstructural characteristics, mechanical properties, and tribological performance of the additively manufactured deposits, a series of characterization procedures was conducted, including Optical Emission Spectroscopy (OES), Optical Microscopy (OM), Scanning Electron Microscopy coupled with Energy-Dispersive X-ray Spectroscopy (SEM/EDS), Vickers hardness testing (HV), and dry sand/rubber wheel abrasive wear testing. The experimental procedures adopted in this study are described in the following sections.

### 2.3. Preparation

The specimens used for characterization were extracted transverse to the deposition direction and prepared according to conventional metallographic procedures. Initially, sectioning was performed using a water-cooled metallographic cutting machine (Struers, MESOTOM, Milton, Australia) to minimize microstructural alterations caused by heat generation during cutting. After sectioning, the specimens were mounted in resin to facilitate handling and provide adequate stability throughout the preparation process.

Subsequently, the specimens were subjected to sequential grinding using silicon carbide (SiC) abrasive papers with grit sizes of 80, 120, 200, 320, 400, 600, and 1200, followed by polishing with a 1 μm alumina suspension until a homogeneous surface free from preparation-induced deformation was obtained. Samples intended for microstructural characterization were chemically etched with Vilella’s reagent for 5 s to reveal the microstructural constituents and phase boundaries.

The surfaces prepared for Optical Emission Spectroscopy (OES) were mechanically ground to remove oxides, surface contaminants, and irregularities resulting from the manufacturing process. The specimens were then subjected to controlled electrical excitation, promoting the emission of characteristic radiation from the chemical elements present in the material, thereby enabling quantitative compositional analysis.

Specimens for the dry sand/rubber wheel abrasion test were machined to the dimensions specified by ASTM G65 [65] using a ROMI U30 milling machine (ROMI, São Paulo, Brazil). Subsequently, the contact surfaces were ground using a Mello P36 surface grinder (Mello, São Paulo, Brazil) to remove surface irregularities and ensure compliance with the standard requirements. Surface roughness measurements were performed after grinding, confirming an average roughness value below 0.8 μm, as required by ASTM G65.

### 2.4. Optical Emission Spectroscopy (OES)

Chemical composition analysis was performed by optical emission spectrometry (OES) using a BRUKER Q2 ION spectrometer (Billerica, Massachusetts, USA) operating at 400 W for 30 s. Prior to analysis, the specimen was sectioned with a Struers MESOTOM metallographic cutter, machined on a Romi U30 milling machine (Romi, São Paulo, Brazil), and surface-ground using a Mello P36 grinder (Mello, São Paulo, Brazil) to obtain a suitable surface finish and minimize roughness. The spectrometer was previously calibrated using certified reference materials to ensure the accuracy and reliability of the measurements. The analyses were conducted in accordance with the procedures adopted by the Materials Characterization and Development Center (CCDM), following the requirements of ASTM E415 [66], ASTM A751 [67], ASTM E1086 [68], ASTM E1251 [69], and ASTM E2994 [70].

### 2.5. Metallography and SEM/EDS Analysis

The microstructural characterization was carried out by scanning electron microscopy (SEM), energy-dispersive X-ray spectroscopy (EDS), and optical microscopy (OM). SEM and EDS analyses were performed using a Zeiss EVO MA 10 microscope (Zeiss, São Paulo, Brazil) equipped with a tungsten filament and operated at an accelerating voltage of 10 kV. Optical micrographs were obtained with an Olympus SC30 microscope (Olympus, São Paulo, Brazil). Prior to the analyses, the specimens were sectioned using a Struers MESOTOM metallographic cutting machine (Struers, Milton, Australia) and extracted from the deposited region. Sample preparation involved successive grinding with SiC abrasive papers of 80, 120, 200, 320, 400, 600, and 1200 grit sizes, followed by polishing on a Fortel PFL polishing machine (Fortel, Willenhall, UK) using a 1 μm alumina suspension diluted in water. To reveal the microstructural constituents and phases present in the deposited material, the polished surfaces were etched with Vilella’s reagent for 180 s prior to both SEM and OM examinations.

### 2.6. X-Ray Diffraction

X-ray diffraction (XRD) analyses were conducted using a Shimadzu LabX XRD-6100 diffractometer (Shimadzu, São Paulo, Brazil), operating with Cu Kα radiation (λ = 1.5406 Å). Phase identification was achieved by matching the experimental diffraction patterns with reference files from the Crystallography Open Database (COD, 2018) using Match!3 software version 3.5.2.104. The obtained data were further processed in Origin 2019 Professional to generate the final diffractograms and confirm the crystalline phases present in the samples.

### 2.7. Testing Hardness

For metallographic preparation and microhardness evaluation, the specimens were sectioned from the deposited region using a Struers MESOTOM metallographic cutting machine (Struers, Milton, Australia). The samples were extracted along the deposition track and prepared through sequential grinding with SiC abrasive papers of 80, 120, 200, 320, 400, 600, and 1200 grit sizes. Subsequently, polishing was performed using a Fortel PFL polishing machine (Fortel, Willenhall, UK) with a 1 μm alumina suspension diluted in water.

The hardness distribution across the deposited material was assessed by Vickers microhardness testing using a Shimadzu HMV-2TADW microhardness tester (Shimadzu, São Paulo, Brazil). The measurements were carried out in accordance with ASTM E384-22 [71] and ASTM E92-23 [72] standards under a load of 9.807 N (HV1) and a dwell time of 10 s. Indentations were performed at previously selected locations along the cross-section of the samples, covering different deposited layers to evaluate the local mechanical response throughout the build. A spacing of 0.5 mm was maintained between adjacent indentations in order to avoid interactions between plastically deformed regions. The resulting hardness values were used to construct hardness profiles and assess the microstructural heterogeneity associated with the manufacturing process.

### 2.8. Dry Sand/Rubber Wheel Abrasion Testing

The dry sand/rubber wheel abrasive wear test was performed in accordance with ASTM G65. The dry sand/rubber wheel abrasion test apparatus used in this study was designed and manufactured by SATC University and is installed at the University’s Tribology Laboratory. During testing, the abrasive flow rate was maintained at approximately 350 g/min, within the range specified by the standard. The abrasive medium consisted of standardized sand supplied by the Institute for Technological Research (ITP), complying with the requirements of NBR 7214 [73]. A normal load of 130 N was applied to the specimens, while the rubber wheel operated at a rotational speed of 200 rpm for a total test duration of 30 min.

The specimens were produced by depositing material onto SAE 1020 carbon steel substrates measuring 25.4 mm in width, 76.2 mm in length, and 12.7 mm in thickness. Following deposition, the samples were machined using a ROMI U30 milling machine and subsequently surface-ground with a Mello P36 grinding machine. These procedures ensured a flat and homogeneous surface condition suitable for abrasive wear testing. After grinding, the specimens were demagnetized and presented a rough surface of 0.8 μm, which was measured using a Mahr MarSurf GD 25 profilometer (Mahr, São Paulo, Brazil).

## 3. Results

The results obtained in this study enabled the evaluation of the microstructural, mechanical, and tribological behavior of deposits produced by Wire Laser Additive Manufacturing (WLAM) using DUR600 welding wire. The combination of the high energy density provided by the laser heat source and the continuous feeding of the filler material promoted the formation of deposits with high deposition rates and distinctive metallurgical characteristics, which were strongly influenced by the successive thermal cycles inherent to the layer-by-layer manufacturing process.

Within this context, the characterization techniques employed were aimed at establishing correlations between the deposition parameters and the resulting chemical composition, microstructural morphology, hardness distribution, and abrasive wear resistance of the deposited material. These analyses provided a comprehensive understanding of the influence of the WLAM process on the final performance of the DUR600 hardfacing deposits, highlighting the relationships among processing conditions, microstructural evolution, and tribological behavior.

### 3.1. Optical Emission Spectrometry for the Chemical Characterization of DUR600

The chemical analysis performed by Optical Emission Spectroscopy (OES) revealed that the material deposited by the WLAM process using DUR600 filler wire exhibited a chemical composition characteristic of wear-resistant steels, primarily distinguished by its relatively high carbon and chromium contents. These alloying elements play a key role in promoting matrix hardening and the formation of carbide phases, which are commonly associated with enhanced resistance to abrasive wear. The chemical composition measured in the deposited material is presented in Table 3.

### 3.2. Metallography and SEM/EDS Analysis

**Figure 3:**
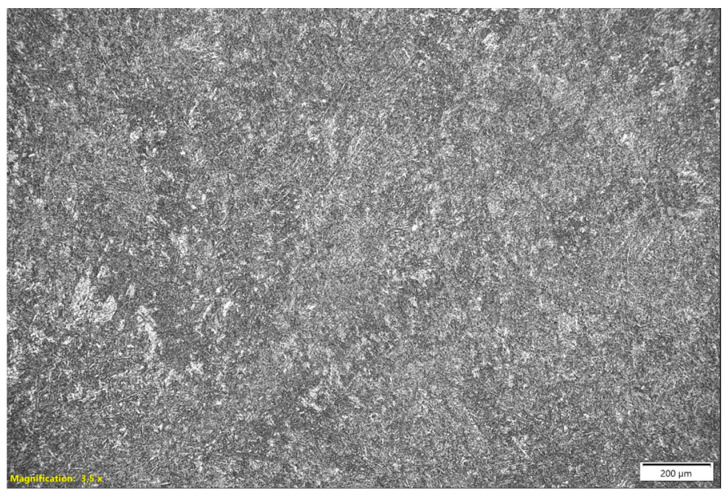
Metallographic analysis of DUR600 produced by Wire and Laser Additive Manufacturing (WLAM) at 100× magnification.

**Figure 4:**
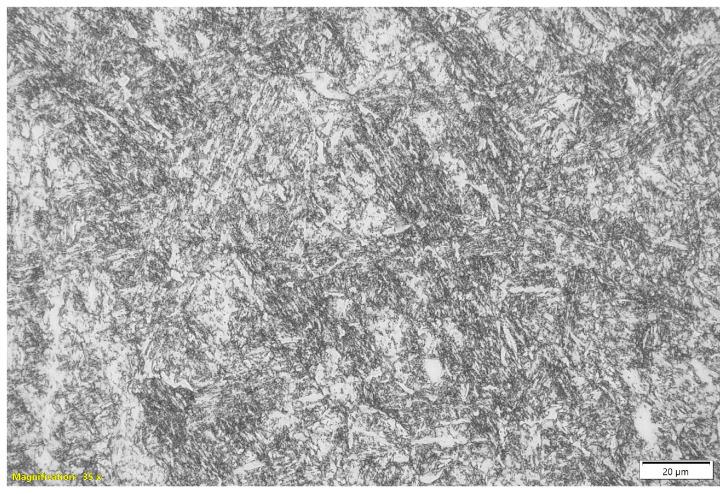
Metallographic analysis of DUR600 produced by Wire and Laser Additive Manufacturing (WLAM) at 1000× magnification.

The metallographic analysis of the sample produced by WLAM using DUR600 filler metal revealed a refined and heterogeneous microstructure, characteristic of the high thermal gradients and rapid cooling rates inherent to the process. The microstructure predominantly consists of a matrix composed of fine acicular structures, relatively homogeneously distributed throughout the analyzed region, indicating rapid solidification of the molten material. Figure 3 shows the metallographic image at 100× magnification, while Figure 4 provides a detailed view at 1000× magnification. According to the figures, a fine martensitic structure is observed, which is typical for steels with this chemical composition.

**Figure 5:**
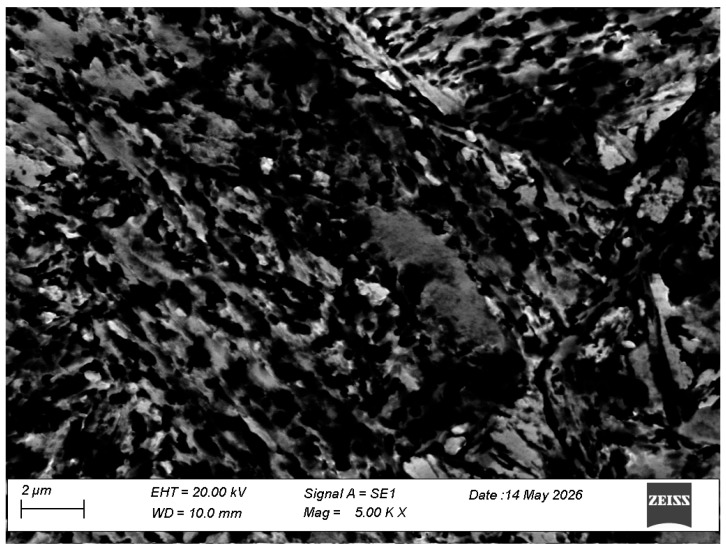
SEM micrograph of the deposited DUR600 material produced by Wire and Laser Additive Manufacturing (WLAM) at 5000× magnification.

The SEM analysis of the sample produced by WLAM using DUR600 filler metal revealed a refined microstructure, characteristic of the high thermal gradients and rapid solidification rates associated with the process. The image shown in Figure 5 presents the microstructure obtained by SEM at 5000× magnification.

**Figure 6:**
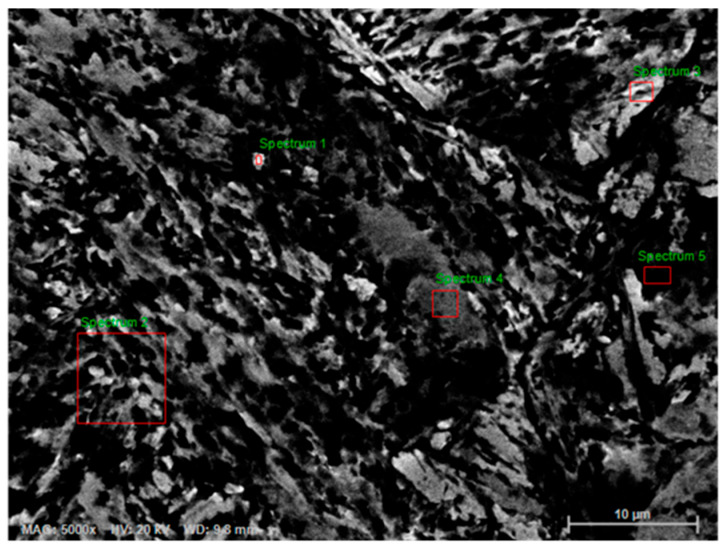
Metallographic analysis of DUR600 produced by Wire and Laser Additive Manufacturing (WLAM) at 5000× magnification, showing the locations selected for EDS analysis.

Using the image obtained by SEM, Figure 6 shows the points where EDS analyses were performed.

The point chemical analysis performed by EDS, in conjunction with SEM, allowed the evaluation of the main chemical elements present in the microstructure of the material deposited by the WLAM process using DUR600 consumable. The spectra obtained from different regions of the sample revealed a relatively homogeneous composition, with a predominance of iron (Fe), as well as significant contents of chromium (Cr) and silicon (Si), consistent with the nominal composition of the filler material. Table 4 presents the EDS values obtained from the points shown in Figure 6.

**Figure 7:**
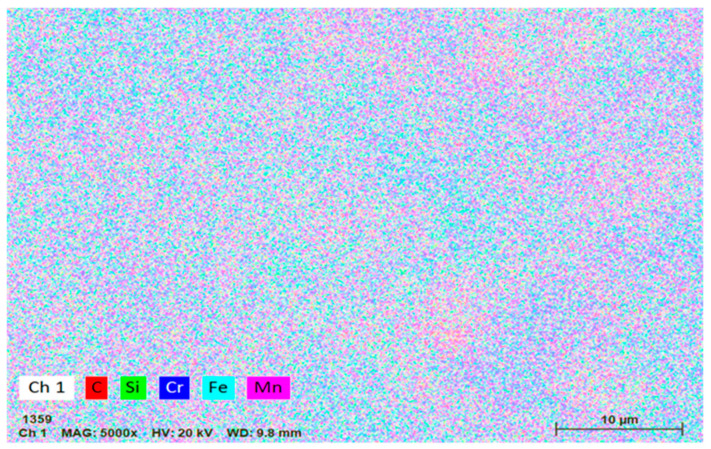
Metallographic analysis of DUR600 produced by Wire and Laser Additive Manufacturing (WLAM) at 5000× magnification, showing the EDS elemental mapping of the main chemical elements.

The elemental mapping by EDS, performed in conjunction with SEM at 5000× magnification, as shown in Figure 7, enabled the evaluation of the spatial distribution of the main chemical elements present in the material deposited by WLAM using DUR600 consumable. The analysis revealed a relatively homogeneous distribution of Fe, Cr, Si, Mn, and C throughout the analyzed region, indicating good metallurgical stability and adequate mixing during the deposition process.

**Figure 8:**
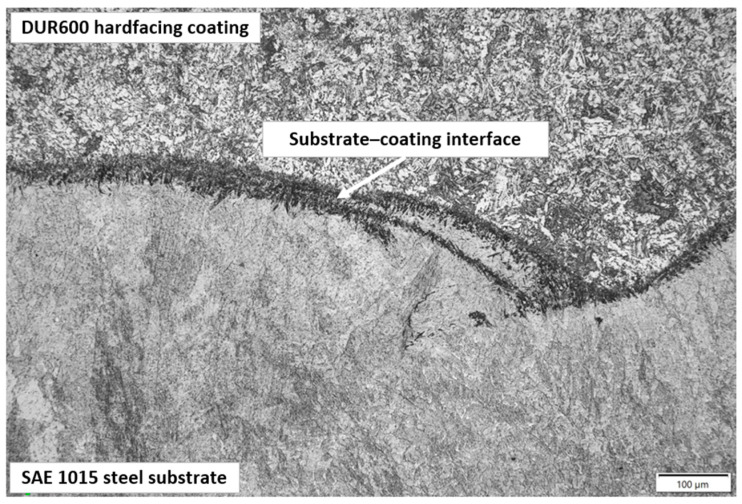
Optical micrograph of the substrate–coating interface between SAE 1015 steel and DUR600 hardfacing coating.

Figure 8 shows the cross-sectional microstructure at the substrate–coating interface. The dark band observed in this region is attributed to the formation of a metallurgical transition zone resulting from substrate dilution and solute redistribution during laser deposition. Partial melting of the SAE 1015 substrate promotes mixing with the DUR600 alloy, generating a composition gradient across the fusion boundary. During directional solidification, chromium and other carbide-forming elements are preferentially rejected into the remaining liquid, leading to localized enrichment near the interface and interdendritic regions. Combined with the high cooling rates characteristic of the laser process, this behavior promotes the formation of a refined martensitic structure containing finely dispersed chromium-rich carbides rather than coarse carbide networks. Therefore, the distinct metallographic contrast observed in this region is associated with chemical segregation and microstructural transformations rather than metallurgical defects. Furthermore, the continuous and crack-free interface indicates effective metallurgical bonding between the SAE 1015 steel substrate and the DUR600 hardfacing coating.

Figure 8 presents an optical micrograph of the interface between the SAE 1015 substrate and the DUR600 hardfacing coating, allowing visualization of the overall morphology of the fusion boundary and the continuity of the metallurgical bond along the deposited layer. The image reveals a well-defined interface with no evidence of macroscopic defects such as cracks, pores, or lack of fusion, indicating adequate process stability and bonding quality. A narrow transition region can also be observed adjacent to the fusion line, resulting from the thermal cycle and partial dilution between the substrate and the deposited material.

**Figure 9:**
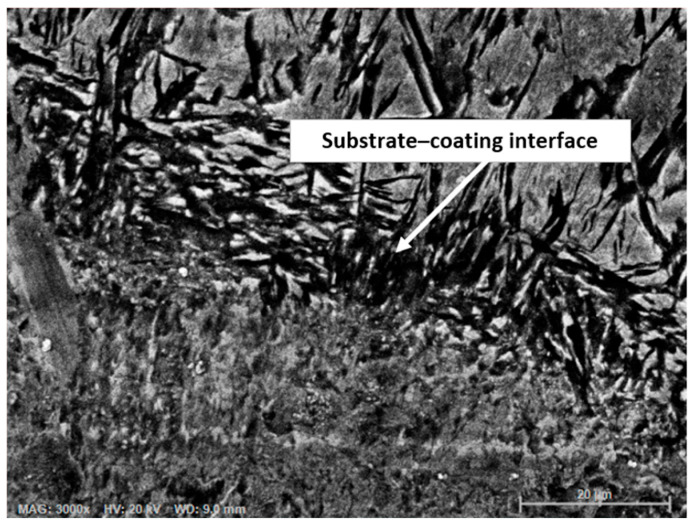
SEM micrograph (3000× magnification) of the substrate–coating interface between SAE 1015 steel and the DUR600 hardfacing coating.

To further investigate the microstructural features of this region, a higher-magnification SEM image was obtained, as shown in Figure 9. The SEM analysis confirms the integrity of the metallurgical bond observed by optical microscopy and reveals the presence of acicular microstructural constituents growing from the substrate–coating interface toward the deposited layer. This morphology is associated with directional solidification under the high thermal gradients characteristic of the WLAM process. Therefore, while the optical micrograph provides a macroscopic view of the fusion boundary, the SEM image enables detailed observation of the microstructural evolution occurring at the interface, demonstrating the effective metallurgical interaction between the SAE 1015 substrate and the DUR600 coating.

**Figure 10:**
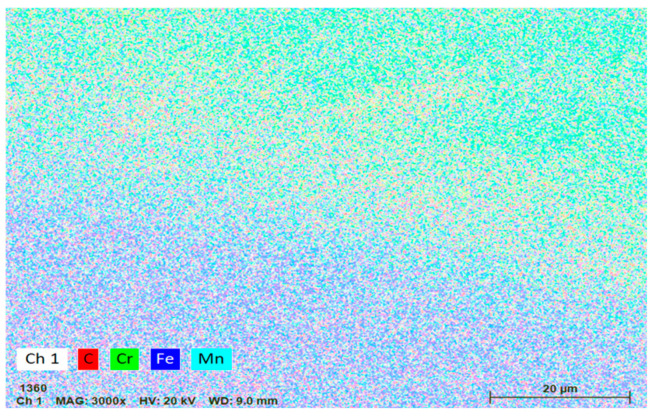
Elemental Mapping by EDS the interface between the SAE 1015 substrate and the DUR600 hardfacing coating at 3000× magnification.

The elemental mapping performed by EDS, carried out in conjunction with SEM at 3000× magnification, as shown in Figure 10, allowed the spatial distribution of the main chemical elements present at the interface between the SAE 1015 steel substrate and the coating deposited using the DUR600 consumable via WLAM to be evaluated. The analysis revealed a homogeneous distribution of Fe, Cr, Mn, and C throughout the analyzed region, indicating good metallurgical stability and adequate mixing during the deposition process.

**Figure 11:**
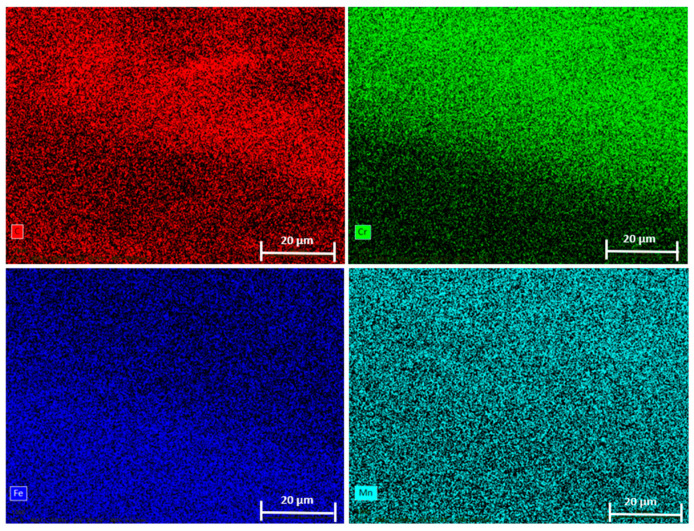
The elemental mapping performed by EDS highlighting separately the elements C, Cr, Fe, and Mn at 3000× magnification.

Figure 11 presents the elemental EDS maps of the interface between the DUR 600 coating and the SAE 1015 substrate. An increase in the intensity of the carbon (C) and chromium (Cr) signals can be observed at the bonding interface. This behavior is associated with the compositional gradient between the two materials and the diffusion phenomena promoted by the high thermal gradients generated during the deposition process. Since the DUR 600 coating contains higher concentrations of carbon and chromium than the SAE 1015 steel, these elements tend to diffuse toward the bonding zone during solidification and cooling.

The local enrichment of carbon and chromium at the interface promotes the formation of a chemical transition zone between the coating and the substrate, contributing to the establishment of an effective metallurgical bond.

The continuous distribution of these elements, without abrupt compositional changes, indicates that the interface does not act as a chemical barrier but rather as a transition zone formed through diffusion and dilution between the coating and the substrate. This behavior confirms the formation of an effective metallurgical bond between the DUR 600 coating and the SAE 1015 substrate, corroborating the microstructural observations obtained by optical microscopy and scanning electron microscopy (SEM).

### 3.3. X-Ray Diffraction

**Figure 12:**
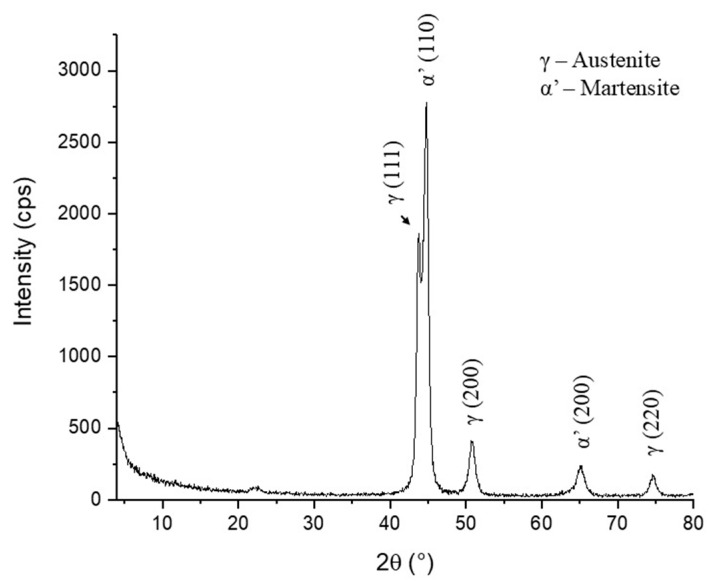
X-ray diffraction (XRD) pattern of the sample, indicating the presence of γ-austenite and α’-martensite phases.

Figure 12 presents the X-ray diffraction (XRD) pattern corresponding to the DUR 600 sample. Qualitative analysis of the diffractogram revealed a multiphase microstructure characterized by the coexistence of the austenitic phase (γ, FCC structure) and the martensitic phase (α’, distorted BCT/BCC structure).

Figure 12 presents the X-ray diffraction (XRD) pattern of the DUR 600 sample. Qualitative analysis of the diffractogram revealed a biphasic microstructure composed of austenite (γ) and martensite (α’). The highest-intensity diffraction peak was identified at approximately 2θ = 44.7°, corresponding to the α’(110) crystallographic plane of martensite, indicating that this phase is predominant under the analyzed condition.

A shoulder is observed adjacent to the main diffraction peak at approximately 2θ = 43.8°, which is attributed to the γ(111) crystallographic plane of austenite. The partial overlap between the γ(111) and α’(110) reflections is characteristic of ferrous alloys containing both martensite and austenite, owing to the close interplanar spacings of these phases. This overlap may also be influenced by residual stresses and lattice distortions within the crystal structure.

The presence of the austenitic phase is further confirmed by the lower-intensity diffraction peaks corresponding to the γ(200) and γ(220) planes, located at approximately 2θ = 50.8° and 74.5°, respectively. Similarly, martensite exhibits a secondary diffraction peak at approximately 2θ = 65.2°, which is indexed to the α’(200) crystallographic plane.

The coexistence of these diffraction peaks confirms that the DUR 600 hardfacing alloy exhibits a microstructure consisting of martensite with a fraction of retained austenite. The significantly higher intensity of the α’(110) peak relative to the austenite reflections indicates that martensite is the predominant phase, whereas the presence of the γ-phase reflections demonstrates that a portion of the austenite remained stable after cooling. This behavior is commonly observed in high wear-resistant alloys due to the influence of alloying elements, particularly carbon and manganese, which promote the stabilization of retained austenite.

### 3.4. Hardness Testing

**Figure 13:**
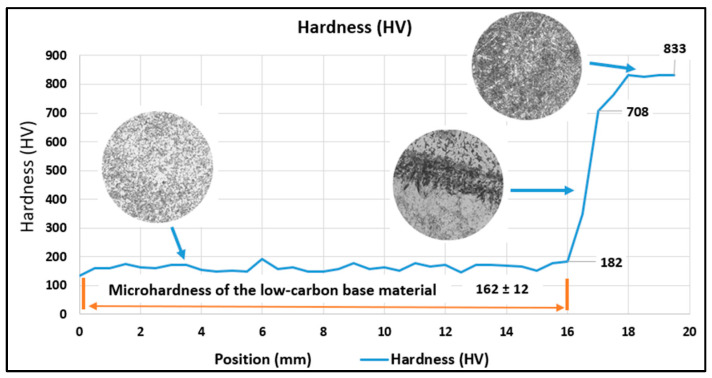
SAE 1015–DUR600 Microhardness Profile.

In order to gain a deeper understanding of the behavior from the base material (SAE 1015) to the hard coating DUR600, a microhardness profile was carried out along the cross-section, as shown in Figure 13. It can be observed that, in the initial region corresponding to the SAE 1015 substrate, hardness values remain nearly constant at approximately 162 ± 12 HV up to about 15 mm. From 16 mm onwards, a progressive increase in hardness is noted, reaching approximately 182 HV, followed by a sharp rise to 708 HV around 17 mm, and culminating in a maximum value of about 833 HV near 18 mm.

This behavior highlights the microstructural transition between the low-carbon steel substrate and the high-hardness DUR600 coating. The micrographs included in Figure 9 clearly illustrate this evolution, correlating the microstructural changes with the corresponding hardness values obtained. The highlighted double arrow marks the region of constant hardness within the substrate, reinforcing the distinction between the base material and the applied coating.

The HV test results performed along the upper layer of the material deposited by WLAM using the DUR600 consumable showed high microhardness values, ranging from 749 HV to 817 HV, with an average of 783 HV ± 18. These results indicate the formation of a highly hardened microstructure, consistent with alloys intended for severe wear applications.

**Figure 14:**
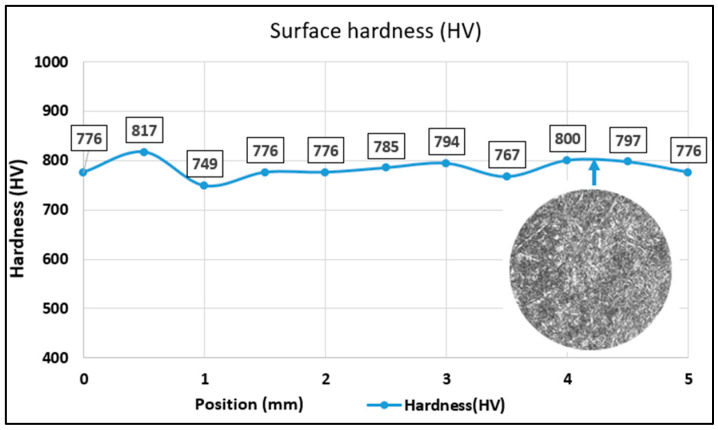
Hardness Profile Graph of the third (3rd) deposited layer, farthest from the SAE 1015 substrate.

After analyzing the data, it was possible to plot a hardness profile, which is shown in Figure 14. The microhardness profile corresponds to the third deposited layer, which is the farthest from the SAE 1015 base material.

### 3.5. Dry Sand/Rubber Wheel Abrasion Testing

**Figure 15:**
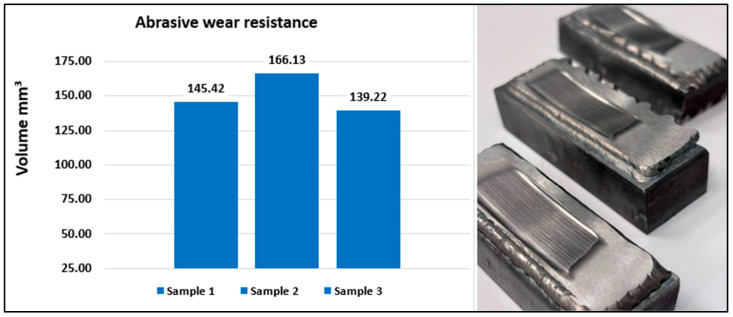
Results of the Rubber Wheel Abrasion Test.

The results obtained from the dry sand rubber wheel abrasive wear test demonstrated that the material deposited by WLAM using the DUR600 consumable exhibited good resistance to abrasive wear. The three evaluated samples showed relatively consistent behavior, with volumetric losses of 145.42 mm^3^, 166.13 mm^3^, and 139.22 mm^3^, resulting in an average volumetric loss of 150.26 mm^3^ after testing. Although some variability was observed among the specimens, the results indicate a stable wear performance and a considerable ability of the deposited material to withstand abrasive conditions. The low volumetric losses obtained suggest that the microstructure generated during the WLAM process contributed positively to the abrasive wear resistance of the deposits. Figure 15 presents the results of the abrasive wear tests and representative specimens after testing.

Table 5 presents a comparison between the hardness and abrasive wear resistance obtained in the present study and those reported in the literature for hardfacing coatings produced using different consumables and deposition processes. The purpose of this comparison is to position the performance of the DUR600 coating produced by WLAM relative to other wear-resistant materials commonly employed in hardfacing applications. The results indicate that the coating developed in this work achieved a hardness level comparable to several high-performance hardfacing alloys, while exhibiting lower abrasive wear resistance than coatings specifically designed for severe wear conditions, such as FeCrB, HCO, and E520 RB. Nevertheless, the volumetric loss of 150.26 mm^3^ was significantly lower than that reported for UTP 7200D deposited by SMAW, demonstrating that the WLAM process is capable of producing coatings with competitive wear performance. These findings highlight the potential of WLAM as an alternative manufacturing route for the production of wear-resistant coatings, while also emphasizing the influence of alloy composition and resulting microstructure on abrasive wear behavior.

**Figure 16:**
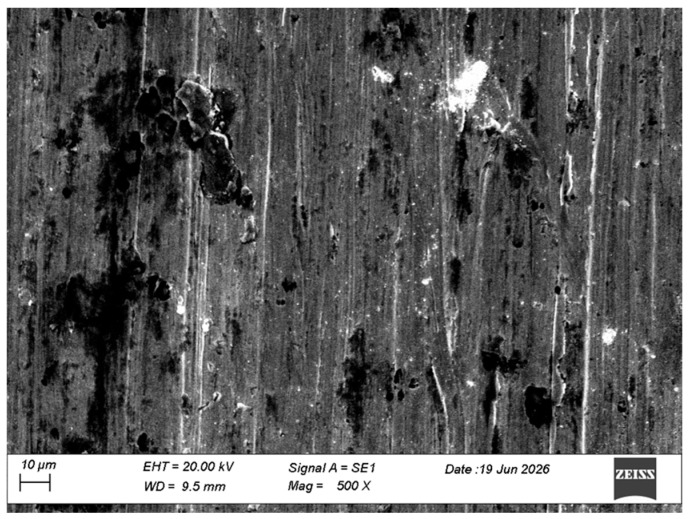
SEM micrograph of the worn surface of the DUR 600 coating after the abrasive wear test at 500× magnification.

Figure 16 presents the SEM micrograph of the DUR 600 coating after the abrasive wear test, acquired at a magnification of 500×. The objective of this analysis was to identify the predominant wear mechanisms acting on the coating surface after abrasion. At this magnification, it is possible to observe the overall morphology of the worn surface, which is characterized by grooves oriented parallel to the sliding direction. These grooves are typical features of abrasive wear and indicate that the abrasive particles displaced across the surface, promoting plastic deformation and material removal.

In addition to the grooves, localized regions exhibiting material accumulation and surface discontinuities can be observed, suggesting the occurrence of localized micro-cutting and material pull-out during the wear process. No evidence of extensive delamination or severe surface cracking was detected, indicating that abrasive wear was the dominant degradation mechanism under the testing conditions.

**Figure 17:**
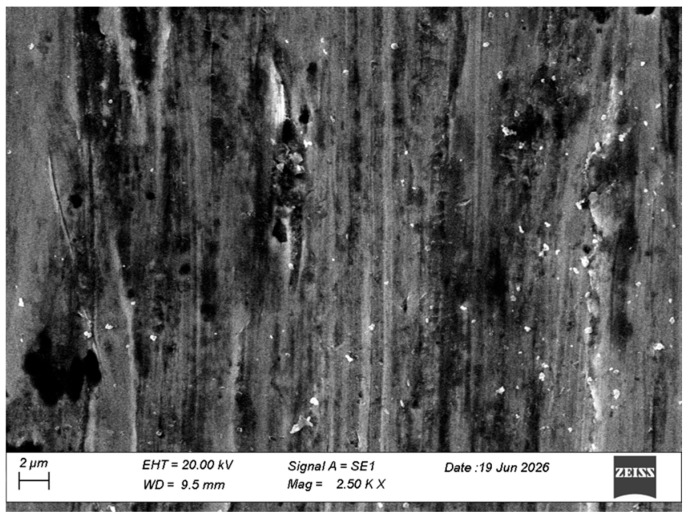
SEM micrograph of the worn surface of the DUR 600 coating after the abrasive wear test at 2500× magnification.

To provide a more detailed evaluation of the wear features, Figure 17 presents a higher-magnification SEM image (2500×) of the worn surface. This higher magnification allows a more detailed examination of the grooves, wear debris, and localized surface damage, enabling a better understanding of the wear mechanisms operating in the DUR 600 coating.

Figure 17 presents the scanning electron microscopy (SEM) micrograph of the DUR 600 coating after the abrasive wear test. The worn surface is characterized by the presence of numerous grooves oriented parallel to the sliding direction, indicating that the predominant wear mechanism was two-body abrasive wear. These grooves were produced by the action of hard abrasive particles, which penetrated the surface, promoting plastic deformation and material removal.

In addition to the continuous grooves, localized regions exhibiting material pull-out and surface discontinuities are observed, suggesting that the abrasive particles induced micro-cutting and localized surface fracture. The coexistence of shallow and deep grooves indicates variations in the penetration depth of the abrasive particles throughout the test, which may be associated with local variations in microstructural hardness and contact conditions.

Small bright particles are also observed dispersed across the worn surface. These features may correspond to wear debris adhered to the surface or fragments of hard phases that remained embedded during the abrasion process. No evidence of severe delamination, extensive cracking, or adhesive wear was identified, indicating that the wear process was predominantly governed by abrasive wear mechanisms.

## 4. Discussion

The obtained composition demonstrates adequate compatibility with applications requiring high surface hardness and abrasive wear resistance. In general, the chemical analysis results confirm that the deposited material exhibits a suitable composition for applications aimed at enhancing surface wear resistance.

According to the manufacturer’s specification data sheet for the DUR 600 wire, referenced as [67], the nominal composition expected for the consumable differs from the final deposited layer. A noticeable reduction was observed: carbon decreased from 0.6 wt.% to 0.424 wt.%, chromium fell from 6 wt.% to 4.134 wt.%, and manganese dropped from 2 wt.% to 0.741 wt.%, whereas the silicon content remained compatible with the original specification. This reduction in alloying elements can be attributed to the chemical dilution with the base material, as well as elemental segregation toward the bonding line, as clearly visualized in Figure 9, Figure 10 and Figure 11.

Associated with the high cooling rates inherent to the laser fusion process, this resulting chemical composition tends to promote the predominant formation of a refined martensitic matrix, a condition often linked to high hardness values and superior tribological performance [33,34,80,81]. Furthermore, studies have shown that the controlled presence of hardening elements such as Cr and Mo contributes to microstructural refinement, increased stability of hardened phases, and improved abrasive wear resistance [33,82].

The relatively limited carbide volume fraction observed in the deposited layer can be attributed to the chemical design of the DUR 600 alloy. With approximately 0.5 wt.% C and 4–5 wt.% Cr, the alloy is intended to produce a hard martensitic/bainitic matrix containing finely dispersed chromium carbides rather than a hypereutectic carbide-rich microstructure. The relatively low carbon content restricts the formation of primary chromium carbides, leaving a significant fraction of chromium in solid solution within the matrix. Furthermore, the high cooling rates associated with laser deposition promote matrix transformation and the precipitation of fine secondary carbides (M_7_C_3_ and M_23_C_6_), instead of coarse carbide networks. Consequently, the resulting microstructure provides a balanced combination of hardness, wear resistance, and impact toughness, which is consistent with the intended application of DUR 600 hardfacing consumables [82,83].

The low levels of impurities observed also indicate good metallurgical quality of the deposited material, promoting greater microstructural homogeneity and reducing susceptibility to the formation of metallurgical defects that could compromise the mechanical performance of the coating.

The observed morphology suggests the predominance of lath martensite and retained austenite, which were confirmed by XRD analysis. This microstructural feature is directly related to the chemical composition of DUR 600, especially the high carbon and chromium contents, which promote high hardenability and the formation of hardened phases during the rapid cooling induced by laser deposition processes [81,82]. The dark regions observed in the micrograph are mainly associated with the martensitic matrix etched by the metallographic reagent [34,84]. The relatively homogeneous distribution of these phases indicates adequate melt pool stability and favorable solidification conditions during deposition, contributing to more uniform mechanical properties throughout the coating [7].

Another important aspect observed is the high level of microstructural refinement. In WLAM processes, the small volume of the melt pool combined with rapid heat extraction results in high cooling rates, promoting intense nucleation and limiting grain growth during solidification [7,85,86,87]. This behavior significantly contributes to increased hardness, mechanical strength, and wear resistance of the deposited material, due to microstructural refinement and the formation of hardened phases associated with the rapid thermal cycles of the process [81,88]. The apparent absence of significant discontinuities, such as high porosity, cracks, or coarse inclusions in the analyzed region, suggests that the processing parameters employed were adequate to obtain a metallurgically stable deposit with good structural integrity. This result is particularly relevant for materials with high carbon equivalent, such as DUR600, since such materials are more susceptible to crack formation induced by residual stresses and high thermal gradients during solidification and cooling [89,90].

The SEM micrograph reveals predominantly fine and interwoven acicular structures, consistent with a refined martensitic morphology, relatively homogeneously distributed throughout the analyzed region. This behavior is directly related to the high carbon and chromium content in DUR600, as well as the high cooling rates imposed by the laser fusion process, which favor non-equilibrium transformations and the formation of refined microstructures containing dispersed carbides [81,87]. The SEM results corroborate the chemical and metallographic analyses, confirming that the combination of the laser fusion process and DUR600 consumable promoted the formation of a predominantly refined martensitic microstructure possibly containing chromium-rich carbides distributed within the matrix. This microstructural condition is desirable for wear-related applications, as it provides high hardness, abrasion resistance, and mechanical stability of the deposited material [82,88]. In addition, the homogeneous distribution of hardening phases observed in the microstructure tends to contribute to a more uniform mechanical response and improved abrasive wear resistance in service.

The SEM micrograph reveals predominantly fine and interwoven acicular structures, consistent with a refined martensitic morphology, relatively homogeneously distributed throughout the analyzed region. This behavior is directly related to the high carbon and chromium content in DUR600, as well as the high cooling rates imposed by the laser fusion process, which favor non-equilibrium transformations. The SEM results corroborate the chemical and metallographic analyses, confirming that the combination of the laser fusion process and DUR600 consumable promoted the formation of a predominantly refined martensitic microstructure possibly containing dispersed carbides. This microstructural condition is highly desirable for wear-resistant applications, as it provides high hardness, abrasion resistance, and mechanical stability of the deposited material [91,92,93].

In general, the EDS results corroborate the metallographic and SEM analyses, confirming the formation of a predominantly martensitic microstructure enriched with carbide-forming elements, especially chromium. This microstructural condition is directly related to the high hardness and wear resistance expected for the DUR600 material deposited by the laser fusion process [91].

Analysis of the interface between the SAE 1015 substrate and the DUR600 coating reveals the formation of a continuous metallurgical bond, free of cracks, pores, or lack-of-fusion regions, indicating adequate process stability during laser deposition. The compositional transition zone observed by optical microscopy, SEM, and EDS is associated with the dilution and diffusion phenomena promoted by the steep thermal gradients characteristic of the WLAM process. During deposition, partial melting of the substrate enables intermixing between the SAE 1015 steel and the DUR600 alloy, promoting redistribution of elements such as Cr and C in the interfacial region. This local enrichment in carbide-forming elements favors the formation of a hardened microstructure near the fusion line, composed of refined martensite and chromium-rich precipitates, thereby contributing to enhanced mechanical and wear resistance in the transition region. The presence of acicular structures oriented from the interface indicates an epitaxial solidification process, in which grain growth occurs preferentially following the crystallographic orientation of the substrate due to the steep thermal gradient imposed by the laser source. The homogeneous distribution of Fe, Cr, Mn, and C observed in EDS mapping confirms that the formed interface does not exhibit an abrupt chemical discontinuity, but rather a gradually modified region resulting from intermixing and diffusion between the two materials. This behavior is desirable in coatings produced by additive manufacturing, as it reduces residual stress concentration and minimizes the likelihood of interfacial failure under mechanical or tribological loading. Recent studies on metallic coatings produced by laser-based processes emphasize that control of thermal cycles and dilution is essential to achieve stable interfaces, given that the elevated cooling rate promotes microstructural refinement and a more uniform distribution of hardening phases. Furthermore, recent work on Fe–Cr–C alloys reinforces that the combination of a martensitic matrix and chromium-rich phases near the interface improves wear resistance by increasing hardness and enhancing the capacity to sustain localized deformation. Collectively, the results obtained demonstrate that the DUR600 coating presented a metallurgically efficient interface with the SAE 1015 substrate, indicating that the parameters employed in WLAM were adequate to promote high adhesion, microstructural control, and potential application in components subjected to severe wear conditions [94,95,96,97].

The hardness profile showed a maximum value of 817 HV at the 0.5 mm position, while the lowest value recorded was 749 HV at the 1 mm position. In general, the values remained relatively homogeneous throughout the analyzed region, with an average of approximately 783 HV ± 18. This low dispersion indicates good metallurgical stability of the deposition process and microstructural uniformity throughout the produced material. The slight oscillation observed between measurement points may be attributed to local microstructural variations resulting from successive thermal cycles characteristic of layer-by-layer additive manufacturing. During deposition, previously solidified regions undergo partial reheating by subsequent layers, which may lead to localized tempering phenomena, grain refinement, and carbide redistribution. These thermal effects can result in small hardness differences between adjacent regions of the deposit [98,99].

To better understand the behavior from the SAE 1015 substrate to the DUR600 hardfacing layer, a microhardness profile was performed across the cross-section, as shown in Figure 10. It can be observed that, in the initial region corresponding to the SAE 1015 substrate, hardness values remain nearly constant at around 162 ± 12 HV up to approximately 15 mm, which is in good agreement with the values reported by other authors [100,101]. From 16 mm onward, a progressive increase in hardness begins, reaching about 182 HV, followed by a sharp rise to 708 HV at around 17 mm, culminating in a maximum value of approximately 833 HV near 18 mm.

This behavior clearly evidences the microstructural transition between the low-carbon steel substrate and the high-hardness DUR600 hardfacing layer. In dissimilar systems produced by welding and hardfacing processes, such hardness gradients are widely reported in the literature and are primarily associated with chemical dilution, heat-affected zone (HAZ) formation, and the development of out-of-equilibrium microstructures such as martensite and carbide-rich constituents in the deposited region [102,103].

According to classical studies on hardfacing and low-carbon steel welding, the substrate region tends to exhibit low hardness variation due to its predominantly ferritic–pearlitic and stable microstructure, which explains the homogeneous behavior observed up to approximately 15 mm [93,104]. The abrupt hardness increase in the intermediate region is associated with the HAZ and the dilution zone, where high cooling rates promote the formation of harder microstructures such as tempered martensite or bainitic constituents, depending on the local chemical composition and thermal cycle imposed by the process [105].

The micrographs presented in Figure 11 clearly illustrate this evolution, correlating microstructural changes with the corresponding hardness values. The observed sharp transition reinforces the strong microstructural gradient typically found in welded and hardfaced systems. The highlighted double arrow delineates the constant hardness region of the substrate, emphasizing the distinction between the base material and the deposited layer.

Abrasive wear test results obtained under dry sand/rubber wheel conditions showed volumetric losses of 145.42 mm^3^, 166.13 mm^3^, and 139.22 mm^3^ for samples 1, 2, and 3, respectively. The relatively small variation among the measured values demonstrates good experimental repeatability and a relatively homogeneous tribological behavior of the deposited material. This low dispersion suggests that the laser fusion process was capable of producing deposits with consistent microstructural characteristics across the analyzed samples, reflecting the thermal stability of the WLAM process and the uniformity of the resulting microstructure.

To provide a comparison with the findings reported in the literature, Wang et al. [106] investigated laser-cladded Ni60 coatings reinforced with different tungsten carbide (WC) contents and evaluated their abrasive wear behavior according to the ASTM G65 standard. The authors reported that increasing the WC content significantly improved wear resistance, with coatings containing 60–80 wt.% WC exhibiting wear rates approximately four times lower than those of conventional hardfacing coatings and nearly six times lower than those of the bucket tooth substrate. According to the authors, this superior performance is attributed to the high hardness and stability of tungsten carbides, which act as barriers against micro-cutting and plastic deformation induced by abrasive particles.

Similar findings were reported by Durmuş et al. [76], who investigated Fe-Cr-C-B hardfacing coatings reinforced with ferroboron and ferrochromium additions. The best wear performance was achieved for the coating containing 40% FeB and 60% FeCr, in which the formation of primary carbides and borides uniformly distributed within a tough matrix significantly reduced material loss during abrasive testing. The authors emphasized that wear resistance is governed not only by coating hardness but also by the morphology, distribution, and volume fraction of hard phases present in the microstructure, which directly influence the material’s ability to resist micro-cutting by abrasive particles [91,107].

In contrast, Casagrande et al. [74] evaluated the UTP 7200D electrode deposited by the SMAW process and reported an average mass loss of approximately 372 mm^3^ in the ASTM G65 wear test. The authors concluded that the material did not exhibit satisfactory performance for predominantly abrasive applications, since its manganese-rich austenitic microstructure was specifically designed to withstand severe impact and compressive loading rather than intense abrasive wear. This characteristic explains its lower wear resistance compared with carbide- and boride-strengthened hardfacing coatings.

Additionally, the findings reported by [79] for Inconel 718 processed via DED with post-treatment by LSP (Laser Shock Peening) revealed a hardness value of 330.9 HV and a volumetric wear loss of only 1.18 mm^3^. A comparison between these data and the results obtained in the present study demonstrates a well-known phenomenon in materials science: high hardness values do not always directly translate into superior abrasive wear resistance. Although the DUR600 coating exhibited significantly higher hardness, its volumetric loss was greater than that of the Inconel 718 alloy. This behavior occurs because tribological performance is governed by a complex synergy of mechanisms, wherein the high toughness, work-hardening capability, and matrix stability of the nickel-based superalloy play a critical role in mitigating wear, thereby outperforming the barriers imposed purely by the bulk hardness of the material.

Overall, although the material developed in the present study exhibited highly reproducible behavior, the measured volumetric losses indicate moderate abrasive wear resistance when compared with conventional hard coatings employed in severe wear applications. This performance can be associated with the nature of the formed microstructure and the distribution of the hardening phases, which, despite contributing to the high hardness values obtained (783 ± 18 HV), were not sufficient to achieve the wear resistance provided by coatings containing high fractions of tungsten carbides or chromium borides [91,107].

The XRD analysis of the DUR600 coating confirms a microstructure composed predominantly of martensite (α’) along with the presence of retained austenite (γ). The predominance of the martensitic phase indicates that a significant transformation occurred during cooling after deposition, imparting high hardness and resistance to plastic deformation—characteristics that are critical for applications subjected to abrasive wear. The presence of retained austenite is associated with the stabilizing effect of alloying elements, such as carbon and manganese, which lower the martensitic transformation start temperature and promote its retention within the microstructure.

This phase combination is beneficial for tribological performance, since martensite acts as the hardening phase, while austenite contributes to matrix toughness and can undergo deformation-induced transformation during wear, promoting surface strain hardening. Conversely, the average wear loss value of 150.26 mm^3^ is also directly associated with the presence of this retained austenite; as a metastable phase with lower hardness than martensite, it can limit the material’s resistance to initial micro-cutting before fully transforming, which justifies the observed volumetric loss. Thus, the microstructure identified by XRD accounts for the wear behavior observed in the DUR600 coating, highlighting the role of the hardness–toughness relationship in governing abrasive degradation mechanisms [94,95].

Analysis of the worn surface of the DUR600 coating revealed that the predominant mechanism during the abrasive test was two-body abrasive wear, characterized by the formation of grooves parallel to the sliding direction, indicating the action of abrasive particles responsible for micro-cutting and localized plastic deformation of the surface. The absence of extensive cracking, delamination, and severe failure demonstrates that the coating exhibited good wear resistance, maintaining surface integrity throughout the tribological loading. The presence of grooves of varying depths suggests local variations in wear resistance, associated with microstructural heterogeneity and the distribution of hardening phases typical of Fe–Cr–C alloys, such as chromium-rich carbides, which act as barriers to abrasive penetration. The bright particles observed may be related to wear debris or fragments of hard phases removed during testing, potentially influencing the evolution of wear through secondary abrasive action. These findings are consistent with recent studies that highlight the influence of microstructure, hardness, and secondary phase distribution on the abrasive behavior of wear-resistant coatings, demonstrating that the performance of DUR600 is associated with the balance between high resistance to abrasive particle penetration and the ability to avoid premature surface fracture [94,95,96,107].

Nevertheless, the results demonstrate that the WLAM-produced DUR600 coating exhibits intermediate wear performance, combining high microstructural uniformity, good deposition process stability, and consistent tribological behavior among the analyzed samples. These findings corroborate the literature by indicating that abrasive wear resistance is controlled not only by hardness but also by the morphology, distribution, and volume fraction of hardening phases within the microstructure. While carbide- and boride-reinforced coatings possess a greater ability to suppress micro-cutting and plastic deformation mechanisms induced by abrasive particles, the DUR600 consumable promotes the formation of a refined martensitic matrix containing chromium-rich carbides, providing a balanced combination of hardness, mechanical stability, and moderate wear resistance. Consequently, this material shows significant potential for additive manufacturing and component repair applications subjected to abrasive conditions, particularly when a compromise between tribological performance, processing simplicity, and manufacturing cost is required.

## 5. Conclusions

The results obtained confirm the technical feasibility of using the WLAM process to deposit hardfacing coatings with the DUR600 consumable onto an SAE 1015 substrate, leading to the following conclusions:

Interfacial Integrity and Transition: The process demonstrated excellent stability, promoting a continuous metallurgical bond free of macrostructural defects. The transi-tion zone revealed a smooth microstructural and chemical gradient driven by dilution and epitaxial solidification phenomena, which is ideal for mitigating residual stresses at the interface.

Uniformity and Hardness Profile: The coating exhibited high elemental homogeneity and metallurgical stability across the deposited layers. The microhardness profile within the deposit region showed low dispersion (average of 783 ± 18 HV), with local fluctua-tions attributed to the successive thermal cycles of reheating and localized tempering inherent to additive manufacturing.

Microstructural Constitution: X-ray diffraction (XRD) analyses confirmed a matrix predominantly composed of refined martensite (α’) along with a fraction of metastable retained austenite (γ).

Tribological Performance and Hardness–Wear Correlation: ASTM G65 tests revealed an average volumetric loss of 150.26 mm^3^ under a predominant two-body abrasive wear mechanism (micro-grooving), with no evidence of premature failure due to embrittle-ment. The wear performance proved to be primarily governed by the hardness of the martensitic matrix rather than by an extensive volumetric reinforcement of carbides.

## Figures and Tables

**Table 1:** Chemical Composition of DUR 600 Hardfacing Electrode [61].

Element	wt.%
Carbon (C)	0.6
Manganese (Mn)	2
Silicon (Si)	1
Chromium (Cr)	6
Iron (Fe)	Balance

**Table 2:** Additive Manufacturing Process Parameters.

Parameters	Configuration
Scanning (mm/s)	300
Amplitude (mm)	2.5
Power (W)	1500
Duty cycle (%)	100
Frequency (Hz)	1000
Wire feed speed (cm/min)	103

**Table 3:** Chemical composition of the part made of DUR600.

Elements	Mass Fraction (%)
Carbon (C)	0.424
Silicon (Si)	1.042
Manganese (Mn)	0.741
Phosphorus (P)	0.0050
Sulfur (S)	0.0041
Chromium (Cr)	4.134
Molybdenum (Mo)	0.461
Nickel (Ni)	0.056
Iron (Fe)	Balance 100

**Table 4:** EDS Data.

Elements	Spectrum 01	Spectrum 02	Spectrum 03	Spectrum 04	Spectrum 05
Iron (Fe)	90.99	88.27	87.61	90.35	86.79
Chromium (Cr)	6.27	7.03	7.54	6.06	7.81
Silicon (Si)	2.74	2.91	2.97	2.73	3.10
Manganese (Mn)	-	0.90	0.90	0.85	1.15
Sulfur (S)	-	0.89	0.97	-	1.15

**Table 5:** Comparison of hardness and abrasive wear resistance of the DUR600 hardfacing coating with similar coatings reported in the literature.

Material	Process	Hardness (HV)	Volume Loss (mm^3^)	Reference
UTP 7200D	SMAW	-	375.3	[74]
DUR600 (Present study)	WLAM	783 ± 18	150.26	Present study
E6-UM-60G	SMAW	538–617	65–68	[75]
FeCrB	Hardfacing	900–1100	25–32	[76]
HCO	SMAW	793	17	[77]
E520 RB	Hardfacing	825	3.31	[78]
INCONEL 718	OL—DED—LSP	330.9	1.18	[79]

## Data Availability

The original contributions presented in this study are included in the article. Further inquiries can be directed to the corresponding author.
